# The relationship between mitochondrial DNA haplotype and the reproductive capacity of domestic pigs (*Sus scrofa domesticus*)

**DOI:** 10.1186/s12863-016-0375-4

**Published:** 2016-05-18

**Authors:** Te-Sha Tsai, Sriram Rajasekar, Justin C. St. John

**Affiliations:** Centre for Genetic Diseases, Hudson Institute of Medical Research, 27-31 Wright Street, Clayton, Vic 3168 Australia; Centre for Genetic Diseases, Department of Molecular and Translational Science, Monash University, 27-31 Wright Street, Clayton, Vic 3168 Australia

## Abstract

**Background:**

The maternally inherited mitochondrial genome encodes key proteins of the electron transfer chain, which produces the vast majority of cellular ATP. Mitochondrial DNA (mtDNA) present in the mature oocyte acts as a template for all mtDNA that is replicated during development to meet the specific energy requirements of each tissue. Individuals that share a maternal lineage cluster into groupings known as mtDNA haplotypes. MtDNA haplotypes confer advantages and disadvantages to an organism and this affects its phenotype. In livestock, certain mtDNA haplotypes are associated with improved milk and meat quality, whilst, other species, mtDNA haplotypes have shown increased longevity, growth and susceptibility to diseases. In this work, we have set out to determine whether mtDNA haplotypes influence reproductive capacity. This has been undertaken using a pig model.

**Results:**

To determine the genetic diversity of domestic pigs in Australia, we have sequenced the D-loop region of 368 pigs, and identified five mtDNA haplotypes (A to E). To assess reproductive capacity, we compared oocyte maturation, fertilization and development to blastocyst, and found that there were significant differences for maturation and fertilization amongst the haplotypes. We then determined that haplotypes C, D and E produced significantly larger litters. When we assessed the conversion of developmentally competent oocytes and their subsequent developmental stages to offspring, we found that haplotypes A and B had the lowest reproductive efficiencies. Amongst the mtDNA haplotypes, the number of mtDNA variants harbored at >25 % correlated with oocyte quality. MtDNA copy number for developmentally competent oocytes positively correlated with the level of the 16383delC variant. This variant is located in the conserved sequence box II, which is a regulatory region for mtDNA transcription and replication.

**Conclusions:**

We have identified five mtDNA haplotypes in Australian domestic pigs indicating that genetic diversity is restricted. We have also shown that there are differences in reproductive capacity amongst the mtDNA haplotypes. We conclude that mtDNA haplotypes affect pig reproductive capacity and can be used as a marker to complement current selection methods to identify productive pigs.

**Electronic supplementary material:**

The online version of this article (doi:10.1186/s12863-016-0375-4) contains supplementary material, which is available to authorized users.

## Background

The maternally inherited mitochondrial genome (mtDNA) is indispensable to the biochemical process of oxidative phosphorylation (OXPHOS) [1], which generates the vast majority of cellular energy (ATP). OXPHOS is conducted in the electron transfer chain and is the only cellular apparatus to have its subunits encoded by the chromosomal and mitochondrial genomes [2]. The pig (*Sus scrofa domesticus*) mitochondrial genome is 16,679 bp in size, double-stranded and circular [3]. It encodes 13 of the 90+ subunits of the electron transfer chain, 22 tRNAs and 2 rRNAs and has one non-coding region, the D-loop, which interacts with nuclear-encoded factors that transcribe and replicate mtDNA [4]. The D-loop also has two hypervariable regions that identify maternal ancestry.

Over billions of years, different maternal lineages have evolved and, based on their mtDNA sequences, they cluster into groupings known as mtDNA haplotypes [5]. Not only do mtDNA haplotypes distinguish between breeds and strains of organisms, they confer advantages and disadvantages on the organism [6, 7]. For example, they influence growth and physical performance in mice [8], adaptation to warm and cold climates [9], and sperm motility [10]. In livestock, they confer economically important traits, such as milk quality [11]. Moreover, it has recently been demonstrated that embryonic stem cell lines possessing the same chromosomal DNA but different mtDNA haplotypes exhibited differential gene expression patterns during cell differentiation [12]. Consequently, it is evident that the mitochondrial genome has an impact on certain cell phenotypes.

Optimization of reproductive traits in livestock worldwide has significant implications for production efficiency and food availability, especially with the ever-increasing population growth, and analysis has primarily been confined to chromosomal effects (see review [13]). In North America, the majority of pig farms report litter size at 11 to 13 piglets and total piglets born alive at 10 to 12 piglets per parity [14]. However, 10 % of farms report litter size at >13 piglets [14] and hyperprolific sows can produce up to 22 piglets per litter [15].

Ovulation rate, uterine efficiency and embryo survival are determiners of the maximal number of piglets produced per parity. Pigs ovulate between 15 to 30 oocytes per estrous cycle [16]. However, litter size is often much lower than the ovulation rate because of prenatal losses of between 30 to 40 % [17]. The quality of the ovulating follicles is highly dependent on the process of follicle development in the preceding period [18], which affects developmental competence. An oocyte that is developmentally competent has the potential to develop post-fertilization. Developmental competence can be determined prior to fertilization by assessing the activity of the enzyme glucose-6-phosphate dehydrogenase (G6PD) at the start of *in vitro* culture [19]. Developmentally competent oocytes suppress the activity of G6PD and cannot reduce the dye brilliant cresyl blue (BCB) and are thus BCB^+^. However, incompetent oocytes continue to express active G6PD and thus reduce BCB (i.e. BCB^−^).

Developmental competence is also linked to mtDNA copy number. BCB^+^ oocytes have > 150,000 copies of mtDNA, progress to metaphase II and develop as embryos once fertilized [20, 21], whereas BCB^−^ oocytes have significantly fewer copies (<100,000) and either fail to fertilize or arrest during pre-implantation development [20, 21]. Indeed, observations in mice [22], cattle [23], pigs [20] and in human [24–26] have demonstrated the importance of mtDNA copy number to fertilization outcome. Supplementation of BCB^−^ oocytes with mitochondria at the beginning of *in vitro* maturation (IVM) results in fertilization rates similar to non-supplemented BCB^+^ oocytes [20]. Consequently, the mtDNA present in the oocyte at fertilization is an investment in developmental outcome, especially as mtDNA replication is only initiated in the embryo proper post-gastrulation once cells commit to a specific fate [27, 28].

To date, the relationship between mtDNA haplotype and reproductive capacity has not been investigated. To achieve this, we have performed in depth analysis of dams representing domestic pigs in Australia. We have sequenced the D-loop region of their mtDNA and determined that they arose from five founder females, which we confirmed through sequencing their whole mitochondrial genomes. Three of the founders originated from Asia and two from Europe. To determine reproductive capacity, we first compared oocyte developmental competence and embryo developmental outcomes. We then observed significant differences in sow litter rates amongst the haplotypes. We further observed haplotype specific differences in reproductive efficiencies during early development. As mtDNA is susceptible to developing sequence variants, the stability of each mtDNA haplotype was determined by assessing the susceptibility of each gene region to the development of mtDNA variants, which, in turn, influenced reproductive capacity. Consequently, each mtDNA haplotype utilizes a different strategy during early development that regulates its respective litter size.

## Methods

All chemicals were obtained from Sigma-Aldrich, St Louis, MO, U.S.A. unless, otherwise, stated.

### Animal ethics statement

Animal ethics committee approval was not required as tissues and ovarian samples were obtained from animals that were slaughtered as part of routine commercial food production. Commercial semen samples used for *in vitro* fertilization (IVF) were purchased from PIC Australia. None of the authors of the present study was involved in the decision to slaughter any animals used in this study.

### Sample collection

Two hundred and sixteen ear-tag tissues from commercial pigs and 152 ovarian tissues from abattoir pigs were collected for mtDNA D-loop sequencing. The samples from commercial pigs originated from two farms in Australia with uniform environmental conditions and were collected without prior knowledge of the animal’s breed. A database search after the construction of the phylogenetic trees revealed that the pigs were Landrace, Large White and Duroc and their cross-breeds. The abattoir pigs originated from various farms in Australia, and samples were collected without the knowledge of the animal’s ID tag or breed. All tissues were stored at -20 °C until DNA extraction was performed. DNA extracted from the 27 ear-tag tissues (see ‘Amplification of the mtDNA D-loop’; haplotype A, *n =* 5; haplotype B, *n =* 7; haplotype C, *n =* 4; haplotype D, *n =* 5; and haplotype E, *n =* 6) was also used to sequence their respective whole mitochondrial genomes and for variant analysis. The entire mitochondrial genomes from denuded oocytes from haplotype D (*n =* 6) were sequenced and also used for variant analysis. MtDNA haplotypes were determined after Brilliant cresyl blue (BCB) staining, IVM and IVF were completed to prevent bias.

### Amplification of the mtDNA D-loop

DNA samples from ear-tag and ovarian tissue were extracted using the Isolate II Genomic Kit (Bioline, London, UK), according to the manufacturer’s protocol. To 200 ng of genomic DNA, 5 μL of 10X NH_4_ Reaction buffer, 1.5 μL of 50 mM MgCl_2_ solution, 0.5 μL of 50 mM dNTPs, 1 μL of 25 μM of each forward and reverse primer (F: 5′-GCATTCCATTCGTATGCAAACC-3′; 5′-ATTGTCGTGCCGGATCATGA-3′), 2.5 U of Bio*Taq* Polymerase™ (Bioline) and 38.5 μL of ddH_2_0 ultrapure water were added. Samples were amplified on a thermocycler (MJ Research DNA Engine PTC 200) at 95 °C for 5 min, and 34 cycles of 94 °C for 30 s, 55 °C for 30 s, 72 °C for 30 s with a final elongation step of 72 °C for 5 min. Products were separated on 2 % agarose gels at 100 V for 60 min, and visualised under UV. Products were purified using the Isolate II PCR and Gel Kit (Bioline), according to the manufacturer’s protocol.

### D-loop sequencing

Each reaction consisted of 50 ng DNA, 1 μL of either 5 μM forward or reverse D-loop primers made up to 16 μL with ultrapure ddH_2_0. The reaction mix was added to the BigDye® Terminator v3.1 Cycle Sequencing Kit (Applied Biosystems, Australia). Sequencing reactions were performed on a 16-capillary 3130xi Genetic Analyzer (Applied Biosystems).

### Phylogenetic analysis

CLC Genomics Workbench (v7.5.1; CLC bio, Aarhus, Denmark) was used to undertake mtDNA sequence alignments for the 368 D-loop sequences and 33 whole mitochondrial genome sequences from different individuals. Firstly, phylogenetic trees were created using the Kimura 2P model [29] and the Neighbor Joining method with 1000 bootstrap replicates. To support this, CLC Genomics Workbench was also used to perform model testing to identify the best model for Maximum Likelihood phylogenetic tree construction. Four different statistical analyses were used: the hierarchical likelihood ratio test; Bayesian information criterion; Minimum theoretical information criterion; and Minimum corrected theoretical information criterion. The models tested were Jukes-Cantor, Felsenstein 81, Kimura 80, HKY and GTR (also known as the REV model). The model deemed the best by most statistical tests was used. For the D-loop sequences, a Maximum Likelihood tree was created using the HKY model [30] and the Neighbor Joining method with 100 bootstrap replicates. The 33 whole mitochondrial genome sequences obtained from next generation sequencing (see section ‘Next Generation Sequencing’) were also used to create a Maximum Likelihood tree using the HKY model and the Neighbor Joining method, with 1000 times bootstrap replicates. To show the relationship between Australian domestic pigs and breeds found elsewhere, the 33 whole mitochondrial genomes from haplotypes A to E (see section ‘Sample collection’) were aligned with 106 whole mitochondrial genome sequences obtained from NCBI Genbank (data obtained on 5th February 2015) and a phylogenetic tree was constructed using CLC Genomics Workbench using the Kimura 2P model and Neighbor Joining method with 1000 bootstrap replicates. To support this, model testing was performed, and the GTR model [31] and the Neighbor Joining method with 100 bootstrap replicates were used to construct a phylogenetic tree. The accession numbers and breed description for the sequences obtained from Genbank are found in Additional file 1 [see Additional file 1].

### Next generation sequencing

Next generation sequencing of whole mitochondrial genomes was performed on amplified mtDNA template obtained by long PCR. 50 ng extracted DNA template was used per reaction with 1× High Fidelity PCR buffer, 100 mM MgSO4, 1 mM dNTPs (Bioline), 1U of Platinum *Taq* High Fidelity (Invitrogen, Carlsbad, CA, USA) and 10 μM of the forward and reverse primers (long1 F: 5′–ATAGGACTCGAACCTAAACCT-3′; long1R: 5′ –GACGAATAGTGCTACGGGAAT-3′; long2 F: 5′-TTCTACCACTACTACTACTGA-3′; long2 R: 5′-AGAATATAGGAGGTTGATGAT-3′). For the amplification of mtDNA from a single oocyte, each oocyte was diluted to a final volume of 50 μL with ultrapure ddH20 and 10 μL was used per reaction. Reaction conditions were 94 °C for 2 min, 35 cycles of 94 °C for 15 s, 60 °C for 30 s and 68 °C for 8 min 45 s. Products were purified using the QIAquick PCR Purification Kit (Qiagen, West Sussex, UK). Purified amplicon pairs generated from long PCR were combined at equal concentrations, prior to the generation of the amplicon libraries. Amplicon libraries were generated using the recommended workflow procedures from the Ion Fragment Library Kit and Ion Xpress™ Template kit (Life Technologies) using 318 chips and run on an Ion Torrent PGM, as described in [32]. > 98 % of the DNA fragments from the library aligned to a pig reference genome (NC_000845.1) The mean coverage depth for the samples was 3395 ± 61 (mean ± SEM). The BAM files are available from the NCBI Sequence Read Archive (Project accession number: SRP059465).

### SNP and variant analysis

Sequences were aligned against the pig mitochondrial genome [3] to generate a representative sequence for each individual. The next generation sequencing data for each individual was then remapped against the representative sequence in order that variant selection could be performed. These tasks were all performed using CLC Genomics Workbench, as described in [32, 33]. For quality control, reads were filtered to exclude those of a nucleotide length of <15 bp, with one nucleotide being trimmed from both ends of each read. All reads accepted into analysis surpassed a Phred quality score of 15. The following parameters were applied to score reads during the selection process for inclusion into the final alignment: a mismatch cost of 2 and an insertion/deletion cost of 3 were set; acceptance of reads that had a minimum of 80 % identity to the reference sequence; exclusion of all duplicate reads; presence of all variants on forward and reverse reads; and a minimum of 50 reads for each variant. A minimum threshold of 3 % was set for a variant to be called. A ‘SNP’ for each mtDNA haplotype was called when all biological replicates from a particular haplotype contained a specific polymorphism unique to that mtDNA haplotype. These polymorphisms occurred at > 90 % frequency in the mitochondrial genome for each individual. ‘Variants’ were determined when a nucleotide change, i.e. single nucleotide variation (SNV), deletion or insertion, occurred at a frequency of 3-49 %, and this varied between individuals from the same haplotype. Therefore, the mean variant load was determined for single variants within each mtDNA haplotype for comparison. The mean number of variants is defined as the total number of variants identified in each of the samples from a specific haplotype and a mean value determined by dividing by the number of samples screened for each haplotype.

### Determining the regions of mtDNA susceptible to the acquisition of variants

To determine the susceptibility of each gene region to the acquisition of variants, we normalized the total number of variants to the size (bp) of each gene region, as described in [32].

### Oocyte collection, BCB selection and *in vitro* maturation

Gilt ovaries were collected from a local abattoir. Follicles of 1–6 mm in diameter were aspirated with an 18 Gauge needle attached to a syringe containing warm Complete Flush handling medium (ViGRO, Pullman, Washington, USA) and cumulus oocyte complexes (COCs) were selected before transfer to Nunc 4 well plates (Thermo Scientific, Roskilde, Denmark) for quality assessment. COCs were incubated in 12 μM BCB in IVM medium (see below) for 60 min at 39 °C, 5 % CO_2_ and in air under oil and selected. BCB^+^ and BCB^−^ COCs were cultured separately in Nunc 4 well plates for 44 to 48 h in pre-equilibrated IVM medium consisting of TCM 199 and 3.05 mM D- glucose, 0.91 mM sodium pyruvate, 0.57 mM Cystein, 10 ng/mL EGF, 10 IU/mL luteinizing hormone (Intervet, Bendigo East, Victoria, Australia), 10 IU/mL follicle stimulating hormone (Intervet, Vic), 75 μg/mL Penicillin, 50 μg/mL Streptomycin and 0.1 % polyvinylalcohol (PVP) at 39 °C, 5 % CO_2_ in air under oil.

### Oocyte and embryo collection for mtDNA copy number analysis

COCs were incubated in handling medium containing 0.2 mg/ml Hyaluronidase for 2 min, followed by repeated pipetting to denude the oocytes. The oocytes were placed in 0.5 % Protease from *Streptomyces griseus* for 30 to 60 s to weaken the zona pellucida followed by thorough washes in Ca^2+^ and Mg^2+^ free Dulbecco's PBS. Individual oocytes were collected in 1.5 mL tubes, and made up to a volume of 50 μL with ddH_2_0 ultrapure water and stored at -20 °C. Oocytes were freeze-thawed twice and vortexed to ensure the homogeneity of the sample.

### *In vitro* fertilization (IVF)

Motile sperm were separated by a 45 %:90 % Percoll gradient in a 15 mL tube by centrifugation at 1500 rpm for 15 min to pellet the sperm. The pellet was washed in Hepes-buffered porcine zygote medium (PZM; see below) and further centrifuged at 1300 rpm for 5 min. The sperm pellet was resuspended in modified Tris-buffered medium (mTBM), and a final concentration of 1x10^6^ mL^−1^ motile sperm was used to co-incubate with the COCs for 4 h in pre-equilibrated mTBM at 39 °C, 5 % CO_2_ in air under oil. The IVF media mTBM consisted of 113.1 mM NaCl, 3 mM KCl, 11 mM Glucose, 7.5 mM CaCl.2H_2_O, 20 mM TRIS, 200 mM sodium pyruvate, 2 mM Adenosine, 20 mM Glutathione and 1 mg/mL BSA.

### Embryo culture

Embryos were denuded and cultured in PZM consisting of 108 mM NaCl, 10 mM KCl, 0.35 mM KH_2_PO_4,_ 0.40 mM MgSO_4._7H_2_O, 25.07 mM NaHCO_3,_ 0.20 mM Sodium Pyruvate, 2.00 mM Ca (Lactate)_2_.5H_2_O, 1 mM L-Glutamine, 5 mM Hypotaurine, 20.00 ml/L BME, 10 mL/L NEAA, 0.065 mg/mL Penicillin G, 0.050 mg/mL Streptomycin and 3 mg/mL BSA. Embryos were cultured at 39 °C, 5 % CO_2_ and 5 % O_2._ Embryos were transferred to fresh, equilibrated PZM at 48 h (day 2) and 120 h (day 5).

### Real time PCR for quantification of mtDNA copy number

Each reaction consisted of 2 μL of template DNA, 10 μL of 2x SensiMix (Bioline), 1 μL of 5 μM of each forward and reverse primer (F: 5′-CTCAACCCTAGCAGAAACCA-3′; R: 5′-TTAGTTGGTCGTATCGGAATCG-3′), and 6 μL of ultrapure ddH20. Reactions were performed in a Rotergene-3000 real time PCR machine (Corbett Research, Cambridge, UK) against a series of 10-fold dilutions (1 ng/μL to 1x 10^-8^ ng/μL) of the target template with the following conditions: 45 cycles of 95 °C for 15 s, 60 °C for 15 s, 72 °C for 15 s. The single peak melt curve was acquired at 75 °C for 15 s.

### Determination of the developmental efficiency

The mean ratios for BCB^+^ to BCB^−^ COCs and maturation, fertilization and blastocyst rates were divided by the mean litter sizes for each mtDNA haplotype to obtain a value for the comparison of conversion rates to an offspring.

### Statistical analysis

All statistical analyses were performed using GraphPad Prism version 6.0d for Mac (GraphPad Software, La Jolla California USA, www.graphpad.com). Quantitative data were tested for normality, and those that followed Gaussian distribution were initially analyzed using one-way ANOVA to test amongst the haplotypes. Those that did not follow Gaussian distribution were analyzed using the Kruskal-Wallis test. If statistical difference was found amongst the haplotypes, further statistical comparisons were made to compare between haplotypes. The litter size data were normally distributed (alpha = 0.05) for each group. Since mtDNA haplotype A had the smallest litter size, each mtDNA haplotype was compared against haplotype A. For qualitative data analysis, Fisher’s exact test was applied. To determine the relationship between mtDNA and reproductive capacity, Pearson correlation with a two-tailed p-value was performed to determine correlation.

## Results

### Five haplotypes identified in Australian domestic pigs

**Fig. 1:**
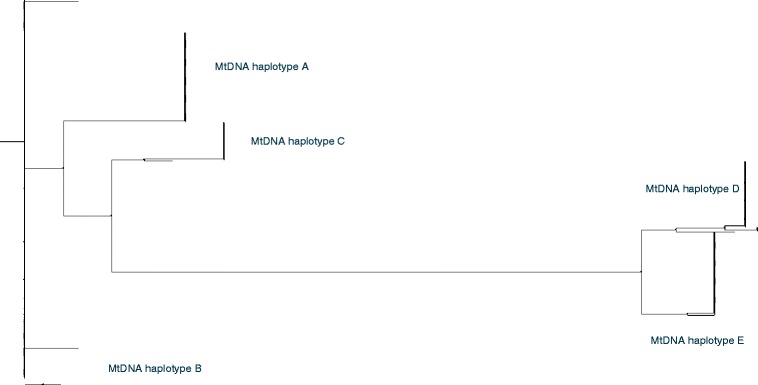
Phylogenetic clustering of mtDNA haplotypes from the D-loop region of 368 gilts and sows representative of commercial pigs in Australia. The phylogenetic tree is constructed by the Kimura 2P model and Neighbor Joining method with 1000 bootstrap replicates. Bootstrap values are expressed as a percentage

**Fig. 2:**
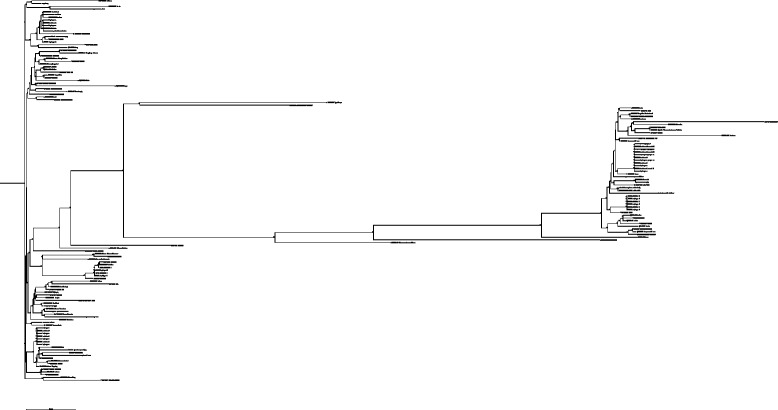
Phylogenetic tree of the whole mitochondrial genomes from haplotypes A to E to validate the D-loop findings. MtDNA from haplotypes A to E were aligned with 106 mtDNA sequences obtained from NCBI to represent pigs from Asia and Europe. The phylogenetic tree is constructed by the Kimura 2P model and Neighbor Joining method with 1000 bootstrap replicates. Bootstrap values are expressed as a percentage. Please zoom in to see annotation in the online version

To determine the degree of mtDNA genetic diversity amongst Australian commercial pigs, we sequenced the D-loop region of the mitochondrial genome of 216 sows from two commercial breeders. Five distinct mtDNA haplotypes were identified, which indicates that each sow is a descendant of one of five common ancestors (Fig. 1). To validate this finding, we sequenced 152 abattoir gilts or sows that were selected randomly to represent multiple Australian farms, and the same five haplotypes were identified (Fig. 1). Additional file 2 shows a phylogenetic tree constructed with the 368 D-loop sequences by Maximum Likelihood [see Additional file 2]. To ensure that the D-loop region outcomes accurately reflected the whole mitochondrial genome for predicting mtDNA haplotypes, we sequenced the entire mitochondrial genomes of 33 pigs by next generation sequencing. Additional file 3 shows a Maximum Likelihood phylogenetic tree constructed with the 33 mitochondrial genomes [see Additional file 3]. MtDNA sequences for haplotypes A to E were clustered with 106 mtDNA sequences obtained from NCBI Genbank to represent pigs from Asia and Europe demonstrating that haplotypes A, B and C were of Asian origin whilst D and E were of European origin (Fig. 2). Additional file 4 shows a phylogenetic tree constructed by Maximum Likelihood to support Fig. 2 [see Additional file 4]. Additional file 1 shows the 106 pig mtDNA sequences and their breed descriptions [see Additional file 1].

A total of 28 polymorphic sites were identified that distinguished the haplotypes (Table 1). Haplotype C possessed the most number of haplotype specific polymorphisms compared with A (13 versus 4), B (13 versus 3), D (13 versus 5) and E (13 versus 3), whilst B and E possessed the fewest (Table 1). Some of these polymorphisms were predicted to induce an amino acid change. For haplotype A, the 8116A > G nucleotide substitution is predicted to induce a Ile to Met amino acid change at position 58 of the ATPase 6 polypeptide (Table 1). Haplotype B possesses a 9758C > A nucleotide substitution, which results in a Leu to Ile amino acid change at position 95 of NADH3 (Table 1). For haplotype C, the unique 13898C > T nucleotide substitution causes a Met to Ile amino acid change at position 64 of NADH6 (Table 1). For haplotype D, there are 4897A > G and 13424 T > C substitutions that lead to a Ile to Val amino acid change at position 330 of NADH2 and a Ile to Thr at position 556 of NADH5, respectively (Table 1). Haplotype E is characterized by 13529 T > C, which leads to a Ser to Phe amino acid change at position 591 of the NADH5 polypeptide (Table 1).

**Table 1:** Unique polymorphic sites that distinguish mtDNA haplotypes A to E

Reference sequence (Accession: AJ002189.1) position	Haplotype A (Accession: KT279758)	Haplotype B (Accession: KT261429)	Haplotype C (Accession: KT279759)	Haplotype D (Accession: KT279760)	Haplotype E (Accession: KT261430)	Overlapping gene	ETC complex	Amino acid change
2309			2309 insT			16S rRNA		
2312		2312 C > T				16S rRNA		
3060				3060 C > T		NADH1	I	
4179			4179 T > C			NADH2	I	
4897				4897 A > G		NADH2	I	CAA05230.1:p. Ile330Val
5585			5585 G > A			COI	IV	
6053			6053 C > T			COI	IV	
6212			6212 C > T			COI	IV	
6359			6359 G > A			COI	IV	
6879		6879 G > A						
7430			7430 A > G			COII	IV	
8116	8116 A > G					ATPase 6	V	CAA05234.1:p.I le58Met
9758		9758 C > A				NADH3	I	CAA05236.1:p. Leu95Ile
10283			10283 T > C			NADH4	I	
10629				10629 C > T		NADH4	I	
12216	12216 G > C					NADH5	I	
13424				13424 T > C		NADH5	I	CAA05241.1:p. Ile556Thr
13529					13529 T > C	NADH5	I	CAA05241.1:p. Ser591Phe
13898			13898 C > T			NADH6	I	CAA05238.1:p. Met64Ile
14378			14378 C > T			CYTB	III	
14594			14594 G > A			CYTB	III	
15212			15212 C > T			CYTB	III	
15615					15615 C > T	D-loop		
15675	15675 T > C					D-loop		
15758				15758 T > C		D-loop		
15840	15840 T > C					D-loop		
15936			15936 A > G			D-loop		
16127					16127 G > A	D-loop		

### Comparison of oocyte developmental competence amongst mtDNA haplotypes

**Fig. 3:**
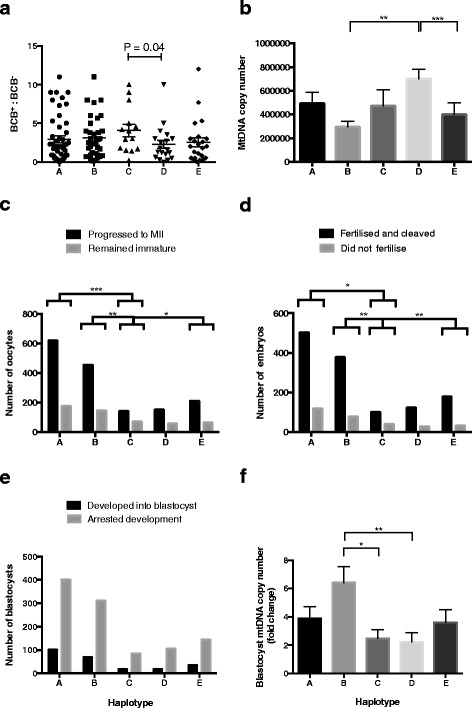
MtDNA haplotypes and developmental rates. **a** Ratios of developmentally competent COCs (mean ± SEM) determined by BCB staining. There were no overall differences between the haplotypes. However, haplotype C produced more developmentally competent COCs than D, determined by *t*-Test. **b** Mean (± SEM) mtDNA copy number for BCB^+^ oocytes. Differences were determined by Kruskal-Wallis test and Dunn’s multiple comparison test. **c** Progression of oocytes to metaphase II following IVM. Haplotypes were compared using Fisher’s exact test. **d** Fertilization rates following IVF. Haplotypes were compared using Fisher’s exact test. **e** Number of blastocysts following 7 days of culture. Haplotypes were compared using Fisher’s exact test. **f** MtDNA copy number fold increase (mean ± SEM) for metaphase II BCB^+^ oocytes to blastocyst. Differences were determined by Kruskal-Wallis test and Dunn’s multiple comparison test. *P < 0.05; **P < 0.01; ***P < 0.001

To determine whether there are differences in oocyte quality amongst the mtDNA haplotypes, we isolated cohorts of developmentally competent COCs from individual ovaries labeled with BCB. The ratio of developmentally competent COCs (BCB^+^:BCB^−^) per ovary (mean ± SEM) for haplotypes A to E were 2.93 ± 0.43, 3.10 ± 0.45, 4.09 ± 0.81, 2.28 ± 0.53, and 2.53 ± 0.58, respectively, which does not represent a significant difference amongst the haplotypes (Fig. 3a). However, haplotype C produced a larger proportion of BCB^+^ oocytes than D when tested in isolation (*P =* 0.04; *t*-Test) (Fig. 3a).

Since mtDNA copy number is also an established indicator of oocyte quality, we quantified mtDNA copy number in BCB^+^ denuded oocytes. Although sows from all haplotypes produced BCB^+^ oocytes with mtDNA copy number that is in the range required for successful fertilization [20], the mean mtDNA copy number for each haplotype was significantly different (*P =* 0.0017). Oocytes from haplotype D possessed significantly more copies of mtDNA (699,867 ± 82,850) than haplotypes B (295,671 ± 45,319; *P <* 0.01) and E (410,301 ± 96,392; *P <* 0.001; Fig. 3b).

### Embryo developmental outcomes amongst mtDNA haplotypes

To determine whether there are differences in embryo developmental competence amongst the mtDNA haplotypes, we assessed oocyte maturation rates for each haplotype and found that the proportion of oocytes progressing to metaphase II following IVM was significantly lower for haplotype C compared with haplotypes A (66 % versus 78 %, *P <* 0.001), B (66 % versus 76 %, *P <* 0.01) and E (66 % versus 76 %, *P <* 0.05) (Fig. 3c). We then inseminated cohorts of BCB^+^ oocytes with fresh boar semen and cultured the embryos to the blastocyst stage. A smaller proportion of oocytes from haplotype C successfully fertilized and cleaved than A (71 % versus 81 %, *P <* 0.05), B (71 % versus 83 %, *P <* 0.01) and E (71 % versus 85 %, *P <* 0.01) (Fig. 3d). However, there were no differences in the rates of blastocyst development amongst the five haplotypes (Fig. 3e). The increase in mtDNA copy number from BCB^+^ oocytes to blastocyst was significantly higher for haplotype B, namely, a 6.4 fold increase compared with increases of 2.5- and 2.2- fold for C (*P <* 0.05) and D (*P <* 0.01), respectively (Fig. 3f).

### The relationship between mtDNA haplotype and litter size

**Fig. 4:**
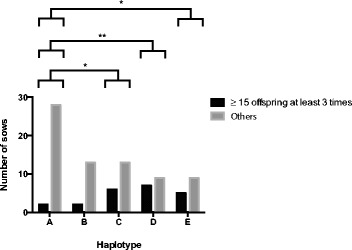
The proportion of sows producing ≥ 15 piglets per parity at least three times. Differences between groups were analyzed using Fisher’s exact test. *P < 0.05; **P < 0.01

In order to determine whether oocyte developmental competence, maturation, fertilization and development to blastocyst affect litter size for each haplotype, we firstly assessed litter size for each haplotype. We assessed the litter sizes of 94 sows that have each had 3 to 10 parities. Since mtDNA is maternally inherited, and the dams had been mated with multiple males, we treated each parity as a separate data point. Additional file 5 shows the spread of breeds across the mtDNA haplotypes [see Additional file 5]. Sows from mtDNA haplotype A produced significantly smaller litter sizes than C (*P <* 0.01), D (*P <* 0.05) and E (*P <* 0.01) (Table 2). Although the number of stillborn per litter was similar amongst the haplotypes (Table 2), the total number of piglets born alive per litter were significantly fewer for haplotype A sows than haplotype C (*P <* 0.01) and E (*P <* 0.01) (Table 2). For each haplotype, we identified the proportion of sows that produced ≥ 15 piglets per parity and had done so at least 3 times. We found that haplotype A had significantly fewer sows that produced ≥ 15 piglets per parity compared with C (*P <* 0.05), D (*P <* 0.01) and E (*P <* 0.05) (Fig. 4).

**Table 2:** Mean (± SEM) litter sizes for haplotypes A to E

Haplotype	Number of sows	Number of litters	Litter size (mean ± SEM)	Number of stillborn (mean ± SEM)	Number of live piglets (mean ± SEM)
A	30	164	10.74 ± 0.28	0.89 ± 0.11	9.85 ± 0.27
B	15	78	11.12 ± 0.41	0.97 ± 0.16	10.14 ± 0.40
C	19	120	12.38 ± 0.34*	1.07 ± 0.15	11.32 ± 0.30*
D	16	101	11.96 ± 0.45**	1.07 ± 0.16	10.89 ± 0.44
E	14	79	12.42 ± 0.40*	0.85 ± 0.15	11.57 ± 0.40*

Statistical differences for each column were determined using ordinary one-way ANOVA followed by parametric multiple comparison against haplotype A

*P < 0.01

**P < 0.05

Having established a relationship between litter size and haplotypes, we then determined the oocyte and embryo conversion rates per offspring for each developmental stage. Haplotype C produced the highest BCB^+^:BCB^−^ ratio but was the least efficient at converting competent oocytes to offspring whilst haplotype D was the most efficient (Table 3). However, haplotype C was the most efficient at converting metaphase II oocytes, based on the number of oocytes that progressed from metaphase II (maturation rate) to offspring, and converting fertilized oocytes to offspring. It was also more efficient than haplotypes A, B and E for the conversion of blastocysts to offspring. Only haplotype D had a better conversion rate from blastocyst to offspring than C. In contrast, haplotypes A and B were the least efficient at converting metaphase II oocytes, fertilized oocytes and blastocysts to offspring. Consequently, we observed differences in the developmental efficiencies amongst the haplotypes.

**Table 3:** Comparison of developmental efficiencies for mtDNA haplotypes A to E

Haplotype	BCB^+^: BCB^−^/Offspring	Maturation (MII) rate/Offspring	Fertilization rate/Offspring	Blastocyst rate/Offspring
A	0.2728	0.0725	0.0753	0.0187
B	0.2779	0.0681	0.0745	0.0162
C	0.3304	**0.0536**	**0.0575**	0.0136
D	**0.1906**	0.0605	0.0677	**0.0116**
E	0.2037	0.0614	0.0684	0.0157

Low index is indicative of high efficiency. Bold indicates the most efficient haplotype for a particular stage of development to generate live offspring

### Characterization of mtDNA haplotypes

**Fig. 5:**
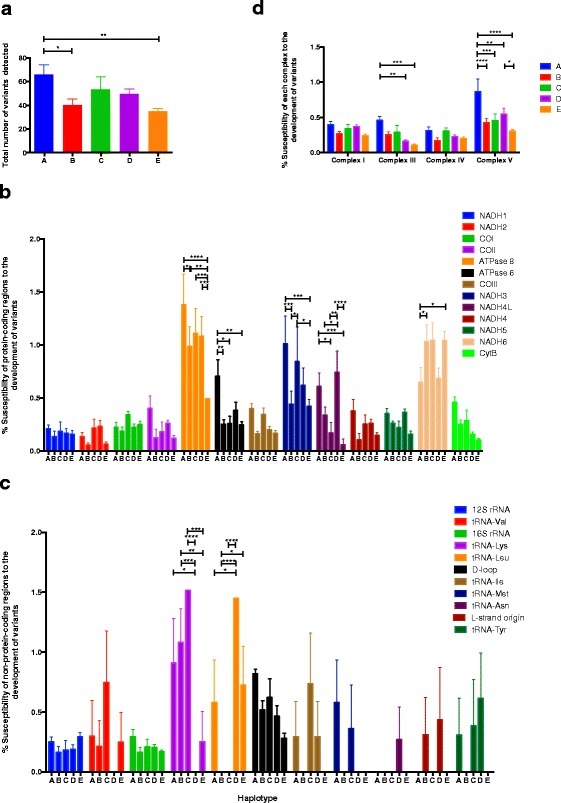
The susceptibility of the mtDNA haplotypes to develop variants identified by next generation sequencing. **a** Total number of variants (mean ± SEM) for haplotypes A to E. **b** Susceptibility of the protein-coding regions to develop variants. **c** Susceptibility of non-protein-coding regions to develop variants. **d** Susceptibility of each electron transfer chain complex to develop variants. Data were analyzed using one-way and two-way ANOVA and Tukey’s multiple comparison test. *P < 0.05; **P < 0.01; ***P < 0.001; ****P < 0.0001

To compare the mtDNA variants harbored by each haplotype, i.e. the presence of SNVs, deletions or insertions occurring at a frequency of 3 to 49 %, we determined the number of variants in each of the ear-tag tissues. Additional file 6 shows the mtDNA variant frequencies for all individuals analyzed [see Additional file 6]. Overall, haplotype A harbored significantly more variants than B (*P <* 0.05) and E (*P <* 0.01) (Fig. 5a). To determine the susceptibility of each gene region to the development of variants, we normalized the total number of variants to the number of base pairs for each gene region. Within the protein-coding regions, ATPase 8, ATPase 6, NADH3, NADH4L and NADH6 showed the most significant variability in frequency amongst the haplotypes (Fig. 5b), whilst in the non-protein-coding regions, tRNA-Lys and tRNA-Leu were most susceptible to gaining variants (Fig. 5c). The combined size for the coding regions for each of the complexes of the electron transfer chain was then determined. Haplotype A was significantly more susceptible to the development of variants than D (*P <* 0.01) and E (*P <* 0.001) for complex III and significantly more susceptible than B (*P <* 0.0001), C (*P <* 0.001), D (*P <* 0.01) and E (*P <* 0.0001) for complex V (Fig. 5d).

**Fig. 6:**
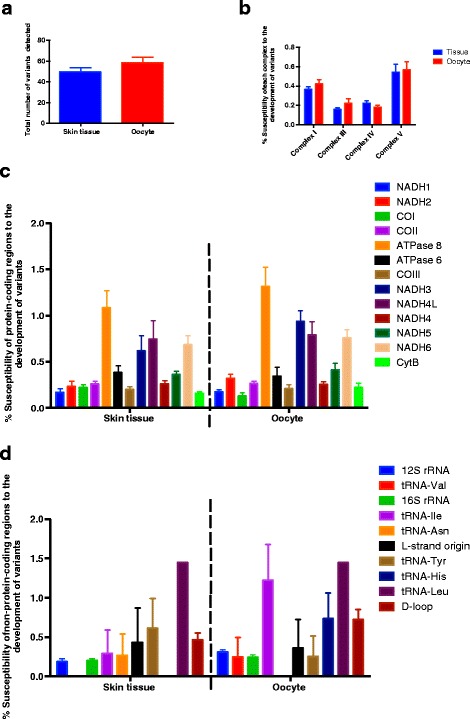
Susceptibility of the mitochondrial genomes from ear-tag tissue samples and oocytes from haplotype D to develop mtDNA variants. Cohorts were compared by one-way and two-way ANOVA. **a** Comparison between ear-tag tissue samples and oocytes for total number of variants. The susceptibility of the electron transfer chain complexes (**b**); the protein-coding regions (**c**); and the non-protein-coding regions (**d**)

Since we hypothesized that mtDNA variants have an impact during periods of high metabolic activity in early development, we sequenced the entire mitochondrial genomes of 6 individual oocytes and ear-tag tissues from haplotype D. The total number of mtDNA sequence variants detected in the ear-tag tissues and oocyte samples was not significantly different (Fig. 6a). In both sample sets, the susceptibility of each complex to the development of variants was also very similar (Fig. 6b). Likewise, for the protein-coding regions, the susceptibility of each gene to developing variants was highly similar (Fig. 6c). Within the non-protein-coding region, tRNA-Val, tRNA-Asn and tRNA-His differed in their susceptibility to the acquisition of variants in both sample sets, but this was not significant (Fig. 6d). This suggests that there is some variation in the segregation of variants.

### Correlation between the levels of mtDNA variants and oocyte developmental competence

**Fig. 7:**
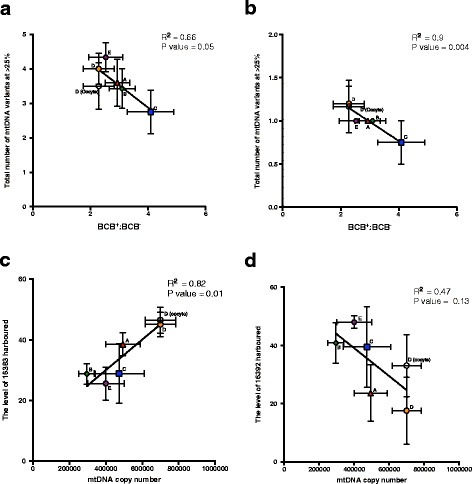
Correlations for mtDNA variants with the ratio of BCB^+^ to BCB^−^ oocytes and mtDNA copy number determined by Pearson’s coefficient. Ear-tag tissue samples from haplotypes A to E are represented by red, green, blue, orange and purple shapes, respectively; open circle represents oocytes from haplotype D. **a** The number of variants harbored by mtDNA at a frequency of > 25 %. **b** Number of variants harbored by complex III (Cyt B) at a frequency > 25 %. **c** Level of variant harbored at position 16383. **d** Level of variant harbored at position 16392

To determine if the number of mtDNA variants identified in the ear-tag tissue samples had an impact on developmental outcome, we used Pearson correlation to assess the relationship between the mean number of mtDNA variants and early developmental stages. Mean number of variants for each mtDNA haplotype was determined by identifying each of the variants present in a particular sample based on next generation sequencing and dividing by the total number of samples analyzed per haplotype. We found that the mean number of variants harbored by each mtDNA haplotype did not correlate with litter size, ratio of BCB^+^ to BCB^−^ oocytes, oocyte maturation, fertilization or blastocyst rates. However, the mean number of variants harbored by each mtDNA haplotype at a frequency of > 25 % correlated negatively with the ratio of BCB^+^:BCB^−^ oocytes (R^2^ = 0.66, *P =* 0.05) (Fig. 7a). Specifically, this correlation was found for complex III (R^2^ = 0.9, *P =* 0.004) but not for I and IV (Fig. 7b). Interestingly, position 14230, located in the complex III coding region, is susceptible to gaining the 14230delC variant at > 25 % frequency for all haplotypes. Moreover, only haplotype D harbored the 14237delA variant at > 25 %. This suggests that the accumulation of Cyt B variants has an impact on oocyte quality during oogenesis. In contrast, variants in complex V were all harbored at less than 15 % frequency, indicating that this complex is only susceptible to developing low levels of variants. This suggests that the regulation of variants is more stringent in complex V.

Since the D-loop region is the binding site for the nuclear-encoded mtDNA replication factors, we hypothesized that the differences in mtDNA copy number amongst the haplotypes are due to polymorphisms in the D-loop. We selected polymorphisms that were harbored by all of the haplotypes and found that mtDNA copy number positively correlated with the level of the 16383delC variant (R^2^ = 0.82, *P =* 0.01) (Fig. 7c). This variant aligned with the conserved sequence box II (CSB II), which is an important region for regulating mtDNA copy number. On the other hand, the level of the 16392 variant, which is located nearby, did not correlate with mtDNA copy number (Fig. 7d).

## Discussion

By sequencing the D-loop region of the mitochondrial genomes from 368 gilts and sows that are representative of Australian commercial pigs, we have identified five mtDNA haplotypes. It is not clear whether other haplotypes existed in the population and were eliminated through their non-commercial viability. Moreover, these data do not include the Australian feral pig population, which may contain other mtDNA genotypes that were introduced into the continent during colonization [34]. Through next generation sequencing, we have determined that haplotypes A, B and C clustered more tightly together and with pigs originating from Asia whilst D and E clustered with pigs originating from Europe. These five haplotypes most likely arose from five common female ancestors that would have been imported to Australia. Based on our sampling of pigs sent to slaughter, it appears that the five mtDNA haplotypes are representative of the core commercial lines and they span across different breeds of pigs. Consequently, one mtDNA haplotype is unlikely to be indicative of a specific breed but, rather, several breeds likely possess the same mtDNA haplotype. In closed populations, such as commercial farms in Australia, where the import of pigs is banned due to biosecurity issues, genetic diversity can be more easily introduced at the chromosomal level through the use of a boar stud or frozen semen. The use of reproductive technology, such as artificial insemination, is especially important for large commercial farms. This is because introduction of live animals into a herd carries a large risk for disease outbreak [35]. On the other hand, as the mitochondrial genome is normally maternally-only inherited, genetic diversity at the mtDNA level is more likely to be restricted to existing lineages.

Most interestingly, our study highlights the effect of mtDNA haplotype on reproductive capacity. Pig litter size is a trait with low heritability ranging between 0.08 and 0.16 [36, 37]. In contrast, the heritability of meat quality is up to 0.75 [38]. Selection for desirable traits in livestock is typically performed using the principles of Mendelian genetics in combination with environmental effects. Traits that are closely related to fitness, such as fertility, are generally known to be less heritable than morphological traits [39, 40]. Here, we report that haplotypes C, D and E have the propensity to generate larger litter sizes than haplotype A. However, when the number of live born offspring was assessed, haplotype D was no longer significant due to higher rates of stillborn, which demonstrates that this haplotype is at maximal production levels considering its higher pre-natal loss rates. Nevertheless, haplotypes C, D and E more consistently produced litters of ≥15 than haplotype A demonstrating the economic importance of these haplotypes.

The increased litter sizes for haplotypes C, D and E are not directly related to the number of developmentally competent oocytes produced by these haplotypes. Whilst haplotype C had a tendency to produce a higher ratio of BCB^+^ to BCB^−^ oocytes than haplotype D, it was evident that a lower proportion of haplotype C oocytes reached maturity and fertilized than the other haplotypes. However, there was no disparity between the haplotypes in terms of blastocyst rates. This suggests that the number of mature oocytes, which are likely those that are ovulated, are not indicative of the number of offspring produced. This is contrary to previous suggestions that follicular development and ovulation rates are indicative of litter size [16–18]. Indeed, direct animal selection for higher ovulation rates has not resulted in increased pig litter sizes [41]. Moreover, we found that fertilization or blastocyst rates were not directly related to litter sizes produced when assessed according to haplotype. Nevertheless, when we compared the developmental efficiencies amongst the haplotypes by determining the conversion rates of oocytes and embryos to an offspring, we found that sows with haplotype C produced the least efficient BCB^+^ oocytes but produced the most efficient metaphase II oocytes and embryos. Only haplotype D produced more efficient blastocysts than C. Overall, haplotypes A and B were the least efficient in all categories.

Our analysis of mtDNA copy number for BCB^+^ oocytes demonstrates haplotype specific copy number. Polymorphisms in the D-loop have been shown to alter mtDNA copy number [42]. In this respect, we have identified a variant at 16383delC that is positively correlated with mtDNA copy number. This variant is located within a highly conserved region known as CSBII. Transcription termination at CSBII results in the production of a primer for mtDNA replication, and this transcript forms a G-quadruplex structure, which interacts with the mitochondrial transcription elongation factor (TEFM) [43–45] to determine if mtDNA is transcribed or replicated [44]. Whilst the overall levels of mtDNA copy number possessed by the oocytes are within previously identified ranges [20, 21], it is evident that levels were highest in haplotype D. Its mean value was at the highest end predicted for successful fertilization outcome suggesting a compensatory mechanism to promote improved developmental outcome. Indeed, all samples sequenced, including oocytes, from haplotype D harbored a Cyt B variant (14237delA) that occurred at > 25 % frequency, which was not found in the other haplotypes, this could explain the large mtDNA copy number possessed by the oocytes from haplotype D. Consequently, there are two regions that could account for differences in mtDNA copy number. We have seen in a previous study that increased levels of naturally occurring mtDNA variants can be compensated for by increased mtDNA copy number [46] and it remains to be determined, which variant, if either, impacts on mtDNA copy number. Nevertheless, it is more likely that the variant in the D-loop region is the most affected site, as this is the site of interaction for the nuclear-encoded transcription and replication factors that translocate to the mitochondrion to drive first transcription and then replication. Sufficient affinity would be required for these processes to be successful.

Moreover, we show that the total number of mtDNA variants that were harbored at > 25 % correlated negatively with the ratio of BCB^+^ to BCB^−^ oocytes. This relationship appears to be specific to the number of variants harbored in Cyt B. This would explain why the BCB^+^ to BCB^−^ ratio is lower for haplotype D compared with C, as those sows would be transmitting high levels of variants to their gametes and produce a larger proportion of oocytes that are not developmentally competent. Although the number of BCB^+^ oocytes did not directly correlate with litter size, it is the quality of oocytes or embryos that determine whether they survive to term. For example, haplotype D may produce fewer BCB^+^ oocytes due to high levels of variants, but a selection (or compensation) mechanism allows haplotype D to produce blastocysts with better conversion efficiency. However, the high rates of stillborn for haplotype D suggest that the persistence of variants can still affect the viability of offspring up to birth. Indeed, amongst other traits, germline inherited mtDNA variants have been shown to have an impact on metabolic activity and litter size in mice [47, 48].

The increase in mtDNA copy number from the BCB^+^ oocyte to the expanding blastocyst stage was the greatest for haplotype B, which suggests that this genome replicates more efficiently during embryo development. It is known that Chinese pig breeds, such as Erhualian and Meishan, are highly prolific and produce larger litter sizes than European and U.S. pig breeds, such as Yorkshire and Landrace [49–51]. This is mainly due to fewer prenatal losses in the Chinese breeds [51, 52]. Numerous investigations have been made to identify the factors affecting embryo survival, which is likely due to maternal environment [49, 53], and/or better embryo quality [50, 54]. Here, we report that the fold change increase in mtDNA copy number for expanding blastocyst stage embryos compared to the metaphase II BCB^+^ oocytes is greatest for haplotype B, which suggests that this genome has a greater propensity for replication at the blastocyst stage when replication is first initiated post-fertilization. Indeed, there appears to be an active process of reducing mtDNA copy number during pre-implantation development where embryo mtDNA is shed into its surrounding environment [55] so that after each cell division the number of mtDNA copies is diluted [21]. At the blastocyst stage, mtDNA replication is restricted to the trophectoderm, whilst replication is suppressed in the inner cell mass to maintain pluripotency [56]. It is likely that the increase in replication efficiency for haplotype B is detrimental to developmental outcome. To this extent, it has recently been shown that, when mtDNA copy number is too high at the blastocyst stage, this leads to aneuploidy [57] and poor implantation outcome [57, 58].

MtDNA turnover has been studied in the context of pig domestication, which is important to understand how modern human society was shaped by agricultural practice [59–61]. The present paper focuses on the distribution of mtDNA haplotypes amongst commercial pigs that were introduced to Australia. Europeans brought domestic pigs to Australia during the 18^th^ and 19^th^ centuries and there is no evidence that Indigenous Australian peoples had any association with pigs [34]. This closed pig population enables us to observe the effect of modern farming practices on mtDNA diversity. Traditionally, mtDNA has been used as a ‘molecular clock’ for tracing maternal lineages. Now, there is accumulating evidence that mtDNA influences cell fate, which in turn is likely to have an impact on the overall phenotype of an animal. For example, we have previously reported that different mtDNA haplotypes matched with the same chromosomal background influenced cellular and lineage specific gene expression patterns [12]. Recent reports have also demonstrated that mtDNA haplotypes influenced metabolic capacity in reconstructed pig cell lines [62]. Hence, we have hypothesized and shown that there are differences in the reproductive capacity of domesticated pigs within commercial herds under the same environmental conditions. However, when subjected to natural environmental conditions, the effects of mtDNA haplotypes on reproductive capacity may play a major role in the establishment and/or dissemination of wild pig populations, as efficient metabolism of food is essential for survival and reproduction.

The dispersal patterns of pigs during the initial stages of their domestication may have also been influenced by the role of mtDNA haplotypes. In Europe, wild pigs were thought to be domesticated approximately 10,000 years before present (BP) based on archaeological evidence of pig morphological changes found in the Fertile Crescent [63]. Based on mtDNA sequences, it has been hypothesized that European wild pigs were hybridized with domesticated Near Eastern pigs during the early domestication processes [61], although more recently, it has been suggested that domestication of the European population occurred continually from local wild pigs [59]. The observation of rapid mtDNA turnover during this period may be due to certain mtDNA haplotypes being preferentially selected based on enhanced breeding efficiencies marked by their propensity to produce larger litters. Furthermore, during the early stages of domestication, offspring with the same mtDNA haplotype could possess either wild or more domesticated features, depending on their chromosomal genes, as we have demonstrated by haplotype not being indicative of breed. Therefore, it is possible that many mtDNA haplotypes were either inadvertently eliminated in the process or preferentially selected, which has led to a rapid turnover in mtDNA. Nevertheless, mtDNA haplotypes are an invaluable source for monitoring genetic selection and predisposition, and domestic pigs today are a result of selection patterns that have arisen since humans transitioned from a hunter-gatherer to an agricultural society.

## Conclusions

In conclusion, we have identified 5 mtDNA haplotypes in the Australian commercial pig population. We observed differences in reproductive strategies for each mtDNA haplotype, and have determined that haplotypes A and B have the lowest reproductive efficiencies. On the other hand, haplotypes C, D and E produced larger litter sizes due to their more efficient reproductive conversion rates. However, haplotype D showed a tendency to produce fewer competent oocytes with higher mtDNA copy number, which is likely due to the mtDNA variants located within the Cyt B gene. We have also shown that the regulation of mtDNA copy number during oocyte maturation is haplotype specific. Additionally, mtDNA copy number is positively correlated with a variant in the D-loop region within the CSB II (16383delC). The differences in reproductive capacities that we have observed may be attributed to a single or a combination of mtDNA polymorphism(s) in the mitochondrial genome. Moreover, the intricate interaction between the mitochondrial and chromosomal genome in each lineage may play a role in these differences [64, 65]. Nevertheless, our results suggest that mtDNA haplotypes influence the reproductive capacity of sows and may influence embryo survival and are indicative of litter size. This highlights the importance of understanding the role of mtDNA haplotypes in livestock breeding programs, and in monitoring genetic selection. Indeed, mtDNA haplotypes could be used to complement current protocols for the selection of economically desirable traits. Equally so, mtDNA haplotypes are important for the management of livestock genetic resources to preserve future traits of interest.

### Ethics

Not applicable.

### Consent to publish

Not applicable.

### Availability of supporting data

The data sets supporting the results of this article are available in the Genbank (Accession numbers KT279758, KT261429, KT279759, KT279760, KT261430) and Sequence Read Archive (Project accession number SRP059465) repositories.

## Additional files

Additional file 1: Breed description for the 106 whole mitochondrial genome sequences obtained from NCBI Genbank (data obtained on 5^th^ February 2015). (DOCX 145 kb) Additional file 2: Phylogenetic clustering of mtDNA haplotypes from the D-loop region of 368 gilts and sows representative of commercial pigs in Australia. The phylogenetic tree is constructed by Maximum Likelihood with the HKY model and Neighbor Joining method with 100 bootstrap replicates. Bootstrap values are expressed as a percentage. (PDF 299 kb) Additional file 3: Phylogenetic clustering of mtDNA haplotypes from 33 whole mitochondrial genome sequences. The phylogenetic tree is constructed by Maximum Likelihood with the HKY model and Neighbor Joining method with 1000 bootstrap replicates. Bootstrap values are expressed as a percentage. (PDF 112 kb) Additional file 4: Phylogenetic clustering of mtDNA haplotypes from 33 whole mitochondrial genome sequences and 106 other whole mitochondrial genome sequences obtained from NCBI Genbank. The phylogenetic tree is constructed by Maximum Likelihood with the GTR model and Neighbor Joining method with 100 bootstrap replicates. Bootstrap values are expressed as a percentage. (PDF 645 kb) Additional file 5: Breed distribution across the mtDNA haplotypes for the 216 commercial pigs. (DOCX 42 kb) Additional file 6: Frequencies of mtDNA variants for all 33 samples sequenced by Next Generation Sequencing. (XLSX 140 kb)

## Abbreviations

ATPase: ATP synthase
ATP: adenosine triphosphate
BCB: brilliant cresyl blue
CO: cytochrome c oxidase
CSB: conserved sequence box
CytB: cytochrome B
D-loop: displacement loop
G6PD: glucose-6-phosphate dehydrogenase
IVF: *in vitro*\ fertilization
IVM: in vitro maturation
MtDNA: mitochondrial DNA
NADH: NADH dehydrogenase
OXPHOS: oxidative phosphorylation
rRNA: ribosomal RNA
TEFM: mitochondrial transcription elongation factor
