# Supplementary material for: The relationship between mitochondrial DNA haplotype and the reproductive capacity of domestic pigs (Sus scrofa domesticus)
Source: BMC Genet. 2016 May 18;17:67. doi: 10.1186/s12863-016-0375-4 (PMC4870755; doi:10.1186/s12863-016-0375-4)
Supplement: Additional file 1: — Breed description for the 106 whole mitochondrial genome sequences obtained from NCBI Genbank (data obtained on 5th February 2015). (DOCX 145 kb) [file 12863_2016_375_MOESM1_ESM.docx]

| **Accession Number** | **Breed** | **Accession Number** | **Breed** |
| --- | --- | --- | --- |
| AB298688 | Ohmini Pig | GQ220329 | Dahe |
| AF034253 | Landrace | GU147934 | Sus scrofa taiwanensis |
| AF304200 | Chinese Meishan | JN601066 | Mangalitsa Blonde breed |
| AF304201 | Italian wild boar | JN601067 | Mangalitsa Blonde breed |
| AF304202 | Landrace | JN601068 | Mangalitsa swallow belly |
| AF304203 | Swedish wild boar | JN601069 | Mangalitsa swallow belly |
| AF486855 | Chinese Zhong Meishan | JN601070 | Meishan |
| AF486856 | Chinese Zang | JN601071 | Meishan |
| AF486857 | Chinese Ningxiang | JN601072 | Turopolje |
| AF486858 | Duroc | JN601073 | Turopolje |
| AF486859 | Chinese Xiang | JN601074 | Yorkshire |
| AF486860 | Chinese Rongchang | JN601075 | Yorkshire |
| AF486861 | Chinese Erhualian | KC250273 | Xiang |
| AF486862 | Chinese Tongcheng | KC250274 | Lantang |
| AF486864 | Chinese Min | KC250275 | Large White |
| AF486865 | Chinese Qingping | KC469586 | Chinese Jinhua pig |
| AF486866 | Landerace | KC469587 | Pietrain |
| AF486867 | Chinese Wuzhishan | KC505406 | Neijiang pig |
| AF486868 | Chinese Yimenghei | KC505407 | Penzhou pig |
| AF486869 | Chinese Diannan Short-ear | KC505408 | Wujin pig |
| AF486870 | Chinese Dahuabai | KC505409 | Yanan pig |
| AF486871 | Chinese Yushanhei | KC505410 | Berkshire |
| AF486872 | Chinese Jiangquhai | KC505411 | Southwest China wild boar |
| AF486873 | Chinese Wannanhua | KF472177 | Shaziling |
| AF486874 | Large White | KF472178 | Ningxiang |
| AP003428 | Large White | KF472179 | Daweizi |
| AY337045 | Duroc | KF569218 | Hybrid of Duroc x Landrace x Yorkshire |
| AY574046 | Hampshire | KF601700 | Taoyuan Black |
| DQ466081 | Nuogu | KF660222 | Qianshao Spotted pig |
| DQ972936 | Type II Lanyu | KF752550 | Yorkshine |
| EF375877 | Type I Lanyu | KF767443 | Wuzhishan |
| EU117375 | Iberian | KF888634 | Bama miniature pig |
| EU333163 | Chinese northeast wildboar | KF971862 | Min |
| FJ236991 | Iberian | KJ720205 | Laiwu Black pig |
| FJ236992 | Iberian | KJ737417 | Ding Yuan |
| FJ236993 | Iberian | KJ737418 | Hang |
| FJ236994 | Iberian | KJ737419 | Huai |
| FJ236995 | Iberian | KJ737420 | Huo shou black |
| FJ236996 | Duroc | KJ737421 | Lai wu |
| FJ236997 | Duroc | KJ737422 | Long lin |
| FJ236998 | European wild boar | KJ737423 | Lu chuan |
| FJ236999 | European wild boar | KJ746662 | Ma shen |
| FJ237000 | European wild boar | KJ746663 | Nei jiang |
| FJ237001 | European wild boar | KJ746664 | Pen zhou shan di |
| FJ237002 | European wild boar | KJ746665 | Ya cha |
| FJ237003 | European wild boar | KJ746666 | Mangalica |
| GQ220328 | Banna Mini | KJ909516 | Wuzhishan |
| KM044239 | Rongchang | KM044240 | Diannan |
| KM073256 | Tibetan | KM094194 | Sandu Black |
| KM101043 | Lantang | KM200762 | Dahuabai |
| KM250424 | Congjiang miniature | KM259826 | Wuyi Black |
| KM275217 | Luchuan | KM433673 | Longlin |
| KP126939 | Bama xiang | KP126954 | Luchuan |
