# Supplementary figures and images for: The relationship between mitochondrial DNA haplotype and the reproductive capacity of domestic pigs (Sus scrofa domesticus)
Source: BMC Genet. 2016 May 18;17:67. doi: 10.1186/s12863-016-0375-4 (PMC4870755; doi:10.1186/s12863-016-0375-4)

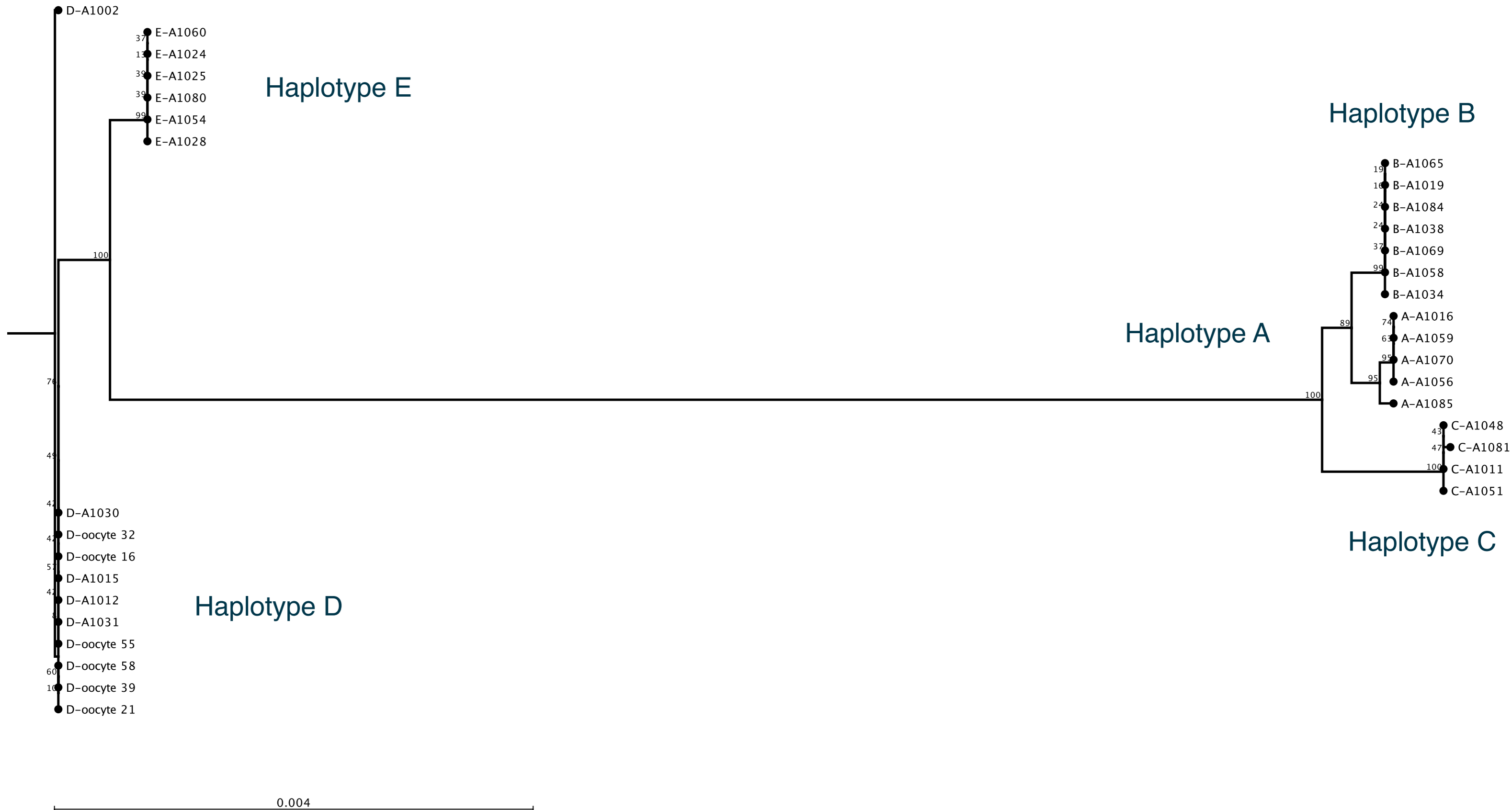

Supplement: Additional file 3: — Phylogenetic clustering of mtDNA haplotypes from 33 whole mitochondrial genome sequences. The phylogenetic tree is constructed by Maximum Likelihood with the HKY model and Neighbor Joining method with 1000 bootstrap replicates. Bootstrap values are expressed as a percentage. (PDF 112 kb) [file 12863_2016_375_MOESM3_ESM.pdf]

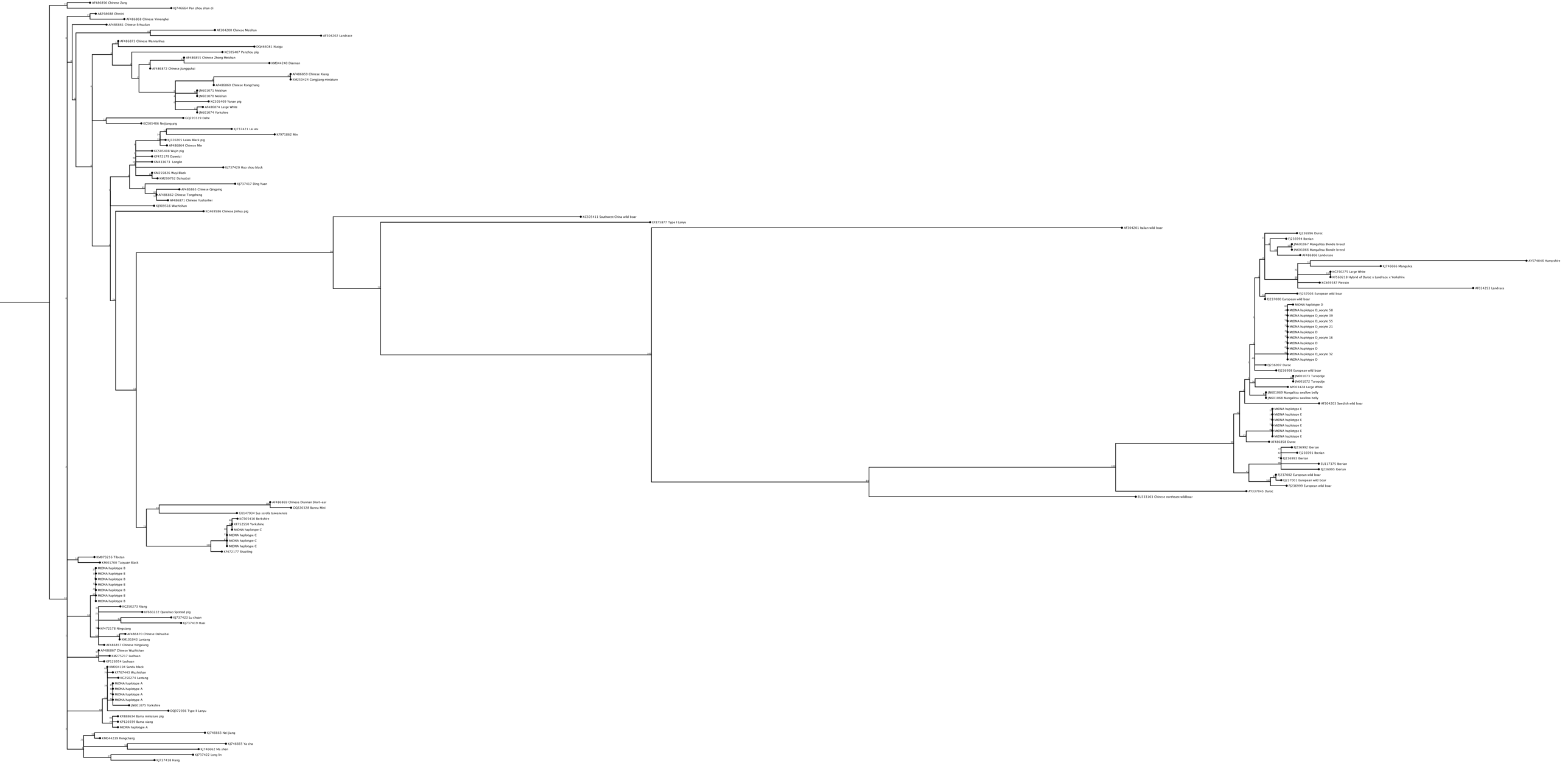

0.001

Supplement: Additional file 4: — Phylogenetic clustering of mtDNA haplotypes from 33 whole mitochondrial genome sequences and 106 other whole mitochondrial genome sequences obtained from NCBI Genbank. The phylogenetic tree is constructed by Maximum Likelihood with the GTR model and Neighbor Joining method with 100 bootstrap replicates. Bootstrap values are expressed as a percentage. (PDF 645 kb) [file 12863_2016_375_MOESM4_ESM.pdf]
