# Supplementary material for: The relationship between mitochondrial DNA haplotype and the reproductive capacity of domestic pigs (Sus scrofa domesticus)
Source: BMC Genet. 2016 May 18;17:67. doi: 10.1186/s12863-016-0375-4 (PMC4870755; doi:10.1186/s12863-016-0375-4)
Supplement: Additional file 5: — Breed distribution across the mtDNA haplotypes for the 216 commercial pigs. (DOCX 42 kb) [file 12863_2016_375_MOESM5_ESM.docx]

| **MtDNA haplotype** | **Large White**  **%** | **Landrace**  **%** | **Duroc**  **%** | **Cross-breed**  **%** |
| --- | --- | --- | --- | --- |
| A | 19.7 | 0 | 60 | 20 |
| B | 73.3 | 0 | 13.3 | 13.3 |
| C | 52.6 | 0 | 47.4 | 0 |
| D | 18.8 | 62.5 | 0 | 18.8 |
| E | 21.4 | 28.6 | 14.3 | 35.7 |
