# Supplementary material for: The relationship between mitochondrial DNA haplotype and the reproductive capacity of domestic pigs (Sus scrofa domesticus)
Source: BMC Genet. 2016 May 18;17:67. doi: 10.1186/s12863-016-0375-4 (PMC4870755; doi:10.1186/s12863-016-0375-4)
Supplement: Additional file 2: — Phylogenetic clustering of mtDNA haplotypes from the D-loop region of 368 gilts and sows representative of commercial pigs in Australia. The phylogenetic tree is constructed by Maximum Likelihood with the HKY model and Neighbor Joining method with 100 bootstrap replicates. Bootstrap values are expressed as a percentage. (PDF 299 kb) [file 12863_2016_375_MOESM2_ESM.pdf]

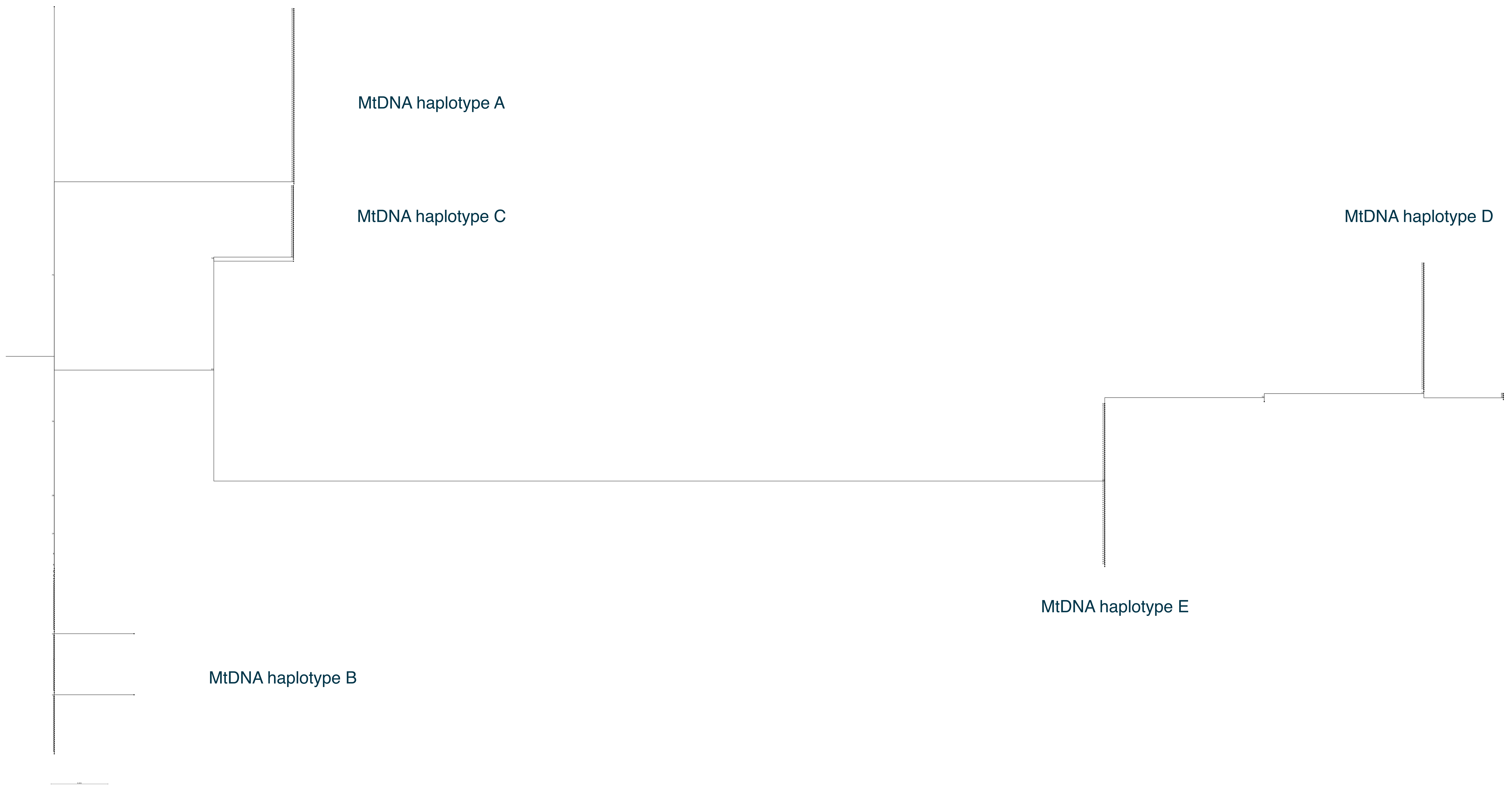
MtDNA haplotype A
MtDNA haplotype C
MtDNA haplotype D
MtDNA haplotype E
MtDNA haplotype B
